# Assessment of medical device features in health technology assessment: a review of NICE medical technology guidance

**DOI:** 10.1017/S026646232510041X

**Published:** 2025-07-29

**Authors:** Stine Pearson, Liana Andrusjaka, Mark Campbell, Cathrine Elgaard Jensen, Henrik Vitus Bering Laursen, Anders Mærkedahl

**Affiliations:** 1 Novo Nordisk, Denmark; 2 GN Hearing A/S, Denmark; 3 Consultant, UK; 4 Department Danish Center for Healthcare Improvements, Aalborg Universitet, Denmark; 5 Department Danish Center for Healthcare Improvements, NHTA, Denmark

**Keywords:** technology assessment, evaluation studies, biomedical technology, health technology, market access

## Abstract

**Objective:**

This investigation evaluates the relationships between claims of patient and health system benefit, evidence in support of those claims, and the recommendation outcomes of medical technologies assessed by the National Institute for Health and Care Excellence (NICE).

**Methodology:**

Data on evidence, claims, and recommendation outcomes were gathered from published Medical Technologies Guidances (MTGs) on the NICE Web site between 1 December 2010 and 11 April 2023. Binary logistic regressions and descriptive data analyses were performed to investigate the correlation between claims, evidence, and recommendation outcomes.

**Results:**

The technology was fully or partially recommended in forty-six (67.7 percent) of sixty-eight MTGs. No correlation was found between types and number of claims and type and quantity of clinical evidence. However, claims supported directly by evidence were significantly correlated (p < 0.016) with recommendation.

**Conclusion:**

Evidence supporting claims is crucial for receiving a full or partial guidance recommendation. There is no clear pattern in what kind of or quantity of evidence leads to a recommendation, and to increase the probability of receiving a favorable recommendation, the manufacturer needs to plan early in the development phases on how to articulate and refine the claims and to substantiate claims through clinical evidence. It is therefore advisable to take advantage of the opportunity for scientific advice, which NICE offers.

## Introduction

Health technology assessment (HTA) of nonmedicine health technologies (e.g., devices, diagnostics, and digital health technologies) is increasingly important internationally in healthcare decision making. In 2009, in the United Kingdom (UK), the National Institute for Health and Care Excellence (NICE) introduced programs that produce evidence-based guidance and advice for healthcare practitioners and managers, and the public (1).

The sponsor of the health technology submits a dossier of information to NICE. A team at NICE evaluates the technology’s eligibility for assessment. The eligibility is defined based on criteria set by NICE, such as the requirement that the technology must be new or an innovative modification of an existing technology. If the technology is deemed eligible, a committee at NICE determines whether it should undergo evaluation and selects the most suitable program for assessment. This study focuses on the Medical Technologies Guidance (MTG) issued by the Medical Technologies Evaluation Programme (MTEP) and the technologies within this framework as, during the time period studied, MTEP evaluated single innovative medical technologies (2;3). After selection, the clinical and economic evidence is appraised. The final MTGs are published on NICE’s Web site, with full or partial recommendations for routine clinical adoption (4;2). If a technology is partially recommended, it is recommended for specific circumstances or populations. The guidance may also recommend that further evidence is needed before routine adoption can be recommended.

## Medical technologies guidance

NICE’s MTEP develops MTGs on single technologies with a cost-saving or cost-neutral value proposition compared with standard care in the UK National Health Service (NHS), based on a submission of clinical and economic evidence from the manufacturer. Companies are invited to describe the claimed patient and healthcare system benefits (claims), and if a product is selected for guidance development, NICE uses these to develop the scope of the evaluation (5;6). The relevance and validity of the company claims and the supporting evidence are critical to the assessment outcome (5). A previous study by Campbell et al. (5) investigated claim types, clinical evidence types, and the outcome of the topic selection decision (whether or not NICE would produce guidance) from 2009 to 2015 and concluded that products with clear claims that are supported by specific evidence are more likely to be selected for guidance development (5). A study by Crispi et al. (2) explored the correlation between different types of studies and the outcomes of MTGs. Their findings indicated that a lower percentage of fully supported MTGs for routine clinical adoption included one or more randomized controlled trials (RCTs) compared to those that were either partially supported or unsupported. Conversely, the statistical analysis revealed no significant difference in the number of randomized and nonrandomized studies between the technologies that were recommended and those that were not recommended (2). In addition, Keltie et al. (7) found that the primary reason for the majority (86.7 percent) of medical technologies not being selected for guidance development was their inadequate demonstration of evidence such as clinical effectiveness and cost benefits. These studies provide important insights for manufacturers who plan to use HTA as part of their market access strategy (2;5;6). We therefore aimed to add insights regarding the factors influencing the recommendation outcome from NICE. Specifically, we sought to develop the work by Campbell et al. (5) by investigating the relationships between claims of patient and health system benefit, evidence in support of those claims, and the recommendation outcome.

## Methods

We analyzed the MTGs published on NICE’s Web site between 10 December 2010 (MTG1) and 11 April 2023 (MTG76). Where an MTG had been reviewed by NICE since the original publication, we used the updated guidance. The review was conducted in three steps: (i) extraction of data on MTGs from NICE’s Web site, (ii) evaluation and organization of the extracted data, and (iii) statistical analysis of the data.

### Data extraction

On 9 July 2023, we searched the NICE Web site (1) for all guidance with “MTG” in the guidance reference number and that was published between 10 December 2010 and 11 April 2023. The data were extracted from both the guidance and the supplementary documents, including the assessment report, final scope, and the sponsor’s submission. A standardized electronic data extraction form was created in Microsoft Excel. All data were extracted by each investigator individually (LA and SP) and thereafter reviewed in collaboration. In case of disagreement regarding data interpretation between the two investigators, each case was discussed until consensus was reached.

### Analytical plan

The guidance outcome was defined in two categories, based on definitions used by NICE: recommended (routine adoption of the technology is fully supported or partially supported) and not recommended (adoption of the technology is not currently supported as the medical technology does not provide significant patient or healthcare system benefits or that further research is recommended before routine adoption can be recommended). Reasons for nonrecommendation were extracted from the committee consideration section of the guidance; in cases with updated guidance, data from the latest version were used in the same manner as in the nonupdated guidance.

From each guidance, the number and type of claims of benefit were collected. The claims of benefits were categorized in three main groups based on categories defined by NICE (8), patient benefits, health system benefits, and sustainability, and further subdivided using the classification described by Campbell et al. (5). Under the patient benefits category, the subcategories include safety, clinical benefits, experiential benefits, psychological benefits, and quicker recovery. Within the health system benefits category, subcategories encompass a shorter length of hospital stay, reduced treatment costs, decreased staff requirements, minimized treatment complications, and an additional category addressing ease of implementation and staff preference. The final category focuses on sustainability, with subcategories involving reduced waste, decreased direct power consumption, and minimized travel. The two subcategories “fewer attendances” (referring to fewer patient visits in the healthcare system) and “lower pay grade of staff” described by Cambell et al. (5) were not included in the current study, as few or no claims were found in these subcategories. An additional category of “other” was added to the health system benefits group, when the claims were not associated with any of the existing categories.

The number of studies for each technology was taken from the description in the guidance and the assessment report; this is based on the studies critically assessed and selected by external assessment centers (EACs) as most relevant to the scope and the highest quality available, based on the type of study. The EAC produces an assessment report with a detailed critique, which forms part of the evidence used in decision making. The clinical evidence was categorized in the following study types, based on what was stated in the specific study: (i) randomized controlled trials (RCTs), (ii) meta-analyses and systematic reviews, (iii) crossover, cohort, and case–control studies, (iv) other studies consisting of nonexperimental, nonspecific observational, and comparative studies, and (v) abstracts and unpublished studies. The MTGs were divided into groups based on the total number of studies included, without regard to study types: one-to-two studies, three-to-five studies, six-to-eight studies, nine-to-eleven studies, and > twelve studies.

Lastly, data were collected about whether the claims of benefit were supported by clinical evidence. In newer MTGs, the guidance and its supplementary assessment report included information about whether the submitted clinical evidence supported the claims. In the assessment report, the claims were reported as being supported, partially supported, or not supported by clinical evidence. However, when this information was not provided, the investigators (LA and SP) assessed the clinical evidence that was provided in the assessment report and the extent to which it supported the claims. Claims were categorized as “supported” when the findings from the clinical study directly supported the claims, for example, that the population, comparator, and outcomes in studies judged to directly support those in the claim.

Claims were “partially supported” when the findings from the clinical study indirectly supported the claim and “not supported” when none of the submitted studies investigated or supported the claim.

### Statistical analyses

A binary logistic regression was performed between the recommendation outcomes (as a dichotomous dependent variable) and the extracted claims and evidence (independent variables), with the outcomes defined as either “recommended” or “not recommended.” This included five logistic regressions with the type of claim, the number of claims, the type of evidence, the quantity of evidence, and the number of claims that were supported, partially supported, and not supported by the clinical evidence. The sample output from the logistic regression is reported as p-values, where significance is assigned when p < 0.05. Further, the odds ratio (OR) including the ninety-five percent confidence interval (CI) is presented. The total count in each category is presented in the sample output. Statistical analyses were performed using Statistical Package for the Social Sciences (SPSS) version 28.

## Results

**Figure 1.**
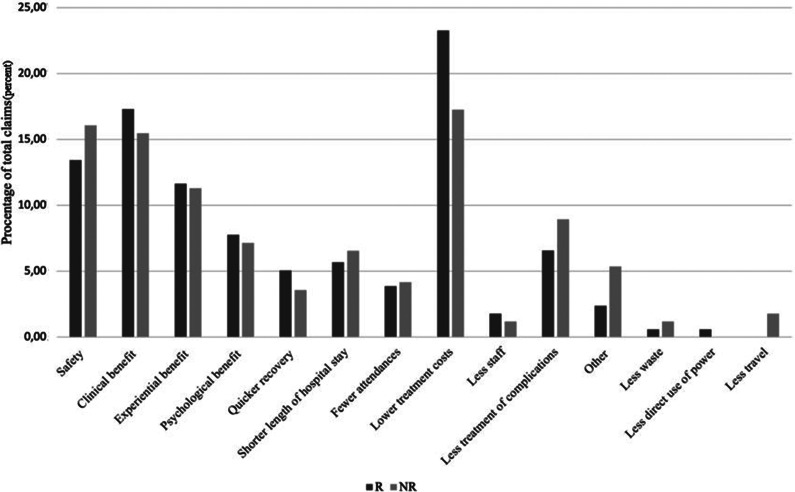
Distribution of claims by recommendation.

Between 1 December 2010 and 11 April 2023, NICE published sixty-eight MTGs of which forty-six (67.6 percent) recommended adoption, whereas twenty-two (32.4 percent) were not recommended. A total of 561 claims were listed in the guidance scopes 8.25 claims per medical technology ranging from three to sixteen. The distribution of claims per category is in Figure 1. The most common claims associated with both recommendation outcomes were lower treatment costs, clinical benefit, and safety, respectively. Of the recommended medical technologies, more than one-fifth (22.1 percent) have claims related to lower treatment cost. The distribution of claims was comparable between the two recommendation groups, except that there were more claims of less travel in the nonrecommended group.

**Figure 2.**
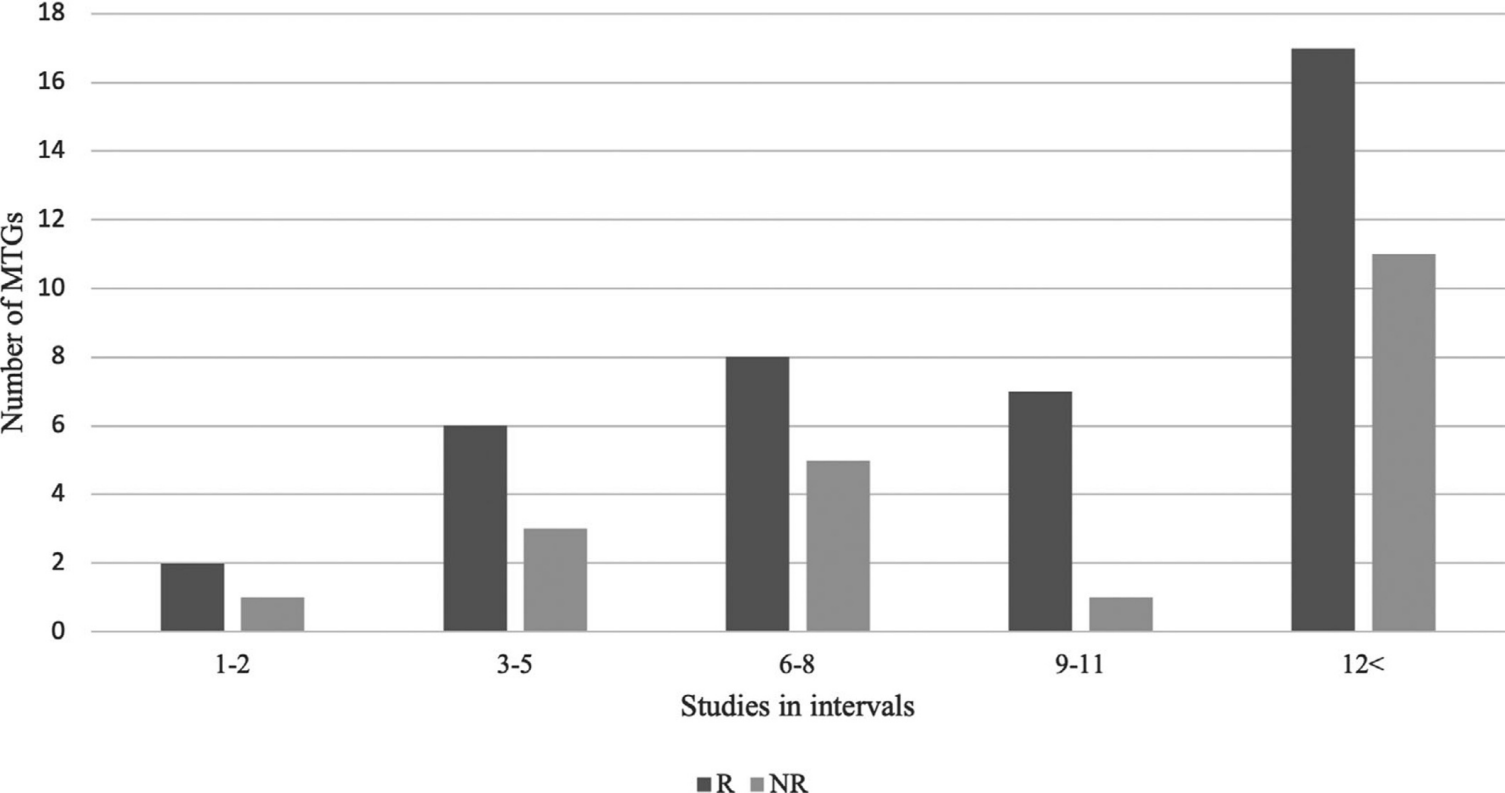
Amount of clinical evidence by recommendation.

The total number of studies across the sixty-eight MTGs were 866, with a mean of 12.7 studies per medical technology ranging from one to thirty-six studies. The quantity of evidence for each of the sixty-eight MTGs is presented in the bar chart in Figure 2. The MTGs were grouped by the quantity of studies in five intervals with one-to-two studies, three-to-five, six-to-eight, nine-to-eleven, and more than twelve studies. For medical technologies with one to two studies, two were recommended and one was not recommended. For most of the evaluated medical technologies, more than twelve clinical studies were submitted.

### Findings of the logistic regression analyses

The logistic regression of claims (Table 1) showed no correlation between the number of claims per technology and the type of recommendation (p = 0.964). The logistic regression of the three principal categories of claims and recommendation outcome showed no correlation with a recommendation: (i) benefits for patients (p = 0.640), (ii) benefits for NHS (p = 0.908), and (iii) sustainability benefits (p = 0.286). There was no evidence of any differences in the ORs (the ninety-five percent CI of all ORs included 1.0).

**Table 1.** Logistic regression of claims

	Variables	R (*n* = 46)	NR (*n* = 22)	Total	p-Value	OR (95 percent CI)
Claims	Patient benefits	210	96	306	0.640	1.077 (0.789–1.469)
	NHS benefits	166	80	246	0.908	0.976 (0.645–1.476)
	Sustainability benefits	4	5	9	0.286	0.575 (0.208–1.590)
	Number of claims^a^	380	181	561	0.964	1.004 (0.843–1.196)

*Note:* The independent variables consist of the number of claims and the three principal claim categories: (i) benefits for patients, (ii) benefits for NHS, and (iii) sustainability benefits. The dependent variable is the recommendation outcome of recommended (R) and notrecommended (NR). The values are illustrated as p-value and OR with ninety-five percent CI. Level of significance was set to p ≤ 0.05.

Abbreviations: R, recommended; NR, not recommended; OR, odds ratio; CI, confidence interval; NHS, National Health Service.

a Number of studies.

The logistic regression of clinical evidence showed no significant correlation between quantity of clinical evidence supporting the MTG and recommendation (p = 0.327). Table 2 illustrates the total lquantity of clinical evidence (*n* = 866). Furthermore, none of the study types were correlated with recommendation: (i) RCT (p = 0.327), (ii) meta-analyses and systematic reviews (p = 0.890), (iii) crossover, cohort, and case–control studies (p = 0.154), (iv) other studies consisting of nonexperimental, observational, and comparative studies (p = 0.891)**,** and (v) abstracts and unpublished studies (p = 0.510). There was no evidence of any differences in the OR (the ninety-five percent CI of all ORs included 1.0).

**Table 2.** Logistic regression of clinical evidence

	Variables	R (*n* = 46)	NR (*n* = 22)	Total	p-Value	OR (95 percent CI)
Clinical evidence	RCTs	148	35	183	0.327	1.093 (0.915–1.306)
	Meta-analyses and systematic reviews	10	4	14	0.890	1.053 (0.517–2.138)
	Crossover, case control, and cohort	128	25	153	0.154	1.007 (0.948–1.402)
	Other types	240	128	368	0.891	1.007 (0.915–1.108)
	Abstracts and unpublished studies	93	55	148	0.510	0.948 (0.807–1.112)
	Quantity of clinical evidence^a^	619	247	866	0.327	1.032 (0.969–1.098)

The independent variables consist of the quantity of clinical evidence and the study types: (i) RCT, (ii) meta-analyses and systematic reviews, (iii) crossover, cohort, and case–control studies, (iv) other studies consisting of nonexperimental, observational, and comparative studies, and (v) abstracts and unpublished studies. The dependent variable is the binary outcome of R or NR. The values are illustrated as p-value and OR with ninety-five percent CI. Level of significance was set to 0.05.

Abbreviations: R, recommended; NR, not recommended; OR, odds ratio; CI, confidence interval; RCT, randomized controlled trial.

a Number of studies.

The logistic regression of the number of claims supported by evidence (Table 3) showed a statistically significant correlation between clinical evidence of any type supporting claims and receiving a full or partial recommendation (p = 0.016). MTGs with claims supported by clinical evidence (ninety-five percent CI: 1.074–1.983) were 1.5 times more likely to be recommended. Further, no correlation was found between the absence of clinical evidence supporting the claims (p = 0.859) or partially supported claims (p = 0.123) and the likelihood of receiving a recommendation.

**Table 3.** Logistic regression of claims supported by evidence

	Variables	R (*n* = 46)	NR (*n* = 22)	Total	p-Value	OR (95 percent CI)
Claims supported by evidence	Supported	225	50	275	0.016^a^	1.459 (1.074–1.983)
	Partially supported	85	91	176	0.123	0.825 (0.647–1.054)
	Not supported	61	39	100	0.859	1.025 (0.780–1.347)

Note: The independent variables consist of the total number of claims supported, partially supported, or not supported by clinical evidence and the dependent variable recommendation outcome of R or NR. The values are illustrated as p-value and OR with ninety-five percent CI. Level of significance was set to 0.05.

Abbreviations: R, recommended; N, not recommended; OR, odds ratio; CI, confidence interval.

a *p*-value within the significance of 0.05.

### The reason for nonrecommendation

In eighty-six percent of the MTGs in which routine adoption was not recommended (twenty-two medical tecknologies), the MTG called for further research, including RCTs or other high-quality comparative evidence. Further real-world evidence (RWE) was recommended in nine percent of cases, and five percent of products were not recommended at all because they were not cost-saving.

## Discussion

This is the largest analysis of claims of benefit and clinical evidence on MTG recommendation outcomes. Overall, 65.7 percent (forty-six medical technologies) of the sixty-eight evaluated MTGs were partially or fully recommended for routine clinical adoption; technologies with claims supported by any quantity or type of specific clinical evidence are 1.5 times more likely to be recommended compared to those with none. No correlation was observed between claim types or number of claims and recommendation; further, no correlation was found between number of clinical studies and recommendation or between study design and recommendation. The impact of claims of benefit and clinical evidence has been studied previously. Campbell et al. (5) found a correlation between some claims and some evidence types on the likelihood of being selected for assessment at NICE (the first evaluation before guidance development). The present study found that the most significant factor for the recommendation decision by NICE is the availability of evidence to support the claims. The results could suggest that the decision-making process is based on the availability of evidence regardless of the study design (as in an evidence hierarchy). For example, some recommended technologies were supported by two or fewer clinical studies, whereas products with twelve or more studies received both recommended and not recommended outcomes.

Campbell et al. (5) found that medical technologies with claims supported by evidence are more likely to be selected for guidance development. The study also reported that medical technologies, which are selected for guidance, had more studies to directly support the claim (5). Further, it is possible that distinct types of claims may necessitate varying types of clinical evidence or that certain claims may be more challenging to substantiate than others. Claim categories such as safety and effectiveness of a medical technology could employ higher quality (based on study type) of evidence, given the importance of these aspects for the well-being of individuals. As in the present study, Crispi et al. (2) investigated the clinical and economic evidence of MTGs between 2009 and 2017 and found no association between study types and recommendation. Their study found that a lower proportion of the recommended MTGs had one or more RCTs, compared to those in partially and not supported MTGs. Their statistical analysis showed no difference in the number of randomized and nonrandomized studies and the recommendation outcome. The findings emphasize that even though RCTs are considered as the gold standard for assessing the effectiveness of healthcare interventions, they do not predict an MTG recommendation (2).

Keltie et al. (7) investigated the same step in the NICE process as Campbell et al. (5) and compared the evidence for products that were selected for guidance development with those that were not assessed for MTG. Overall, the majority (86 percent) of nonprogressed medical technologies were judged to have inadequate demonstration of clinical effectiveness (7). This finding is in line with findings from the reasoning for nonrecommendation in the MTGs found in the present study, where lack of supporting clinical evidence accounted for eighty-six percent (7).

### Strengths and limitations

A strength of this study was the independence of the two investigators who evaluated and characterized the relevant data for analysis. This was done to increase the internal validity of the study and decrease the risk of information bias, also known as measurement bias, as the investigators may influence each other (9). Despite this, the limitations must also be considered. Firstly, the grouping of the claims and clinical evidence was manually performed by the study investigators (LA and SP). This means that the grouping could have been different and perhaps changed the results. However, the groupings were based on the previous analysis by Campbell et al. (5) and the grouping of the clinical evidence was made with consideration of the ranking according to the different levels of evidence. Further, this study was limited by the information that was publicly available on NICE’s Web site. Although the MTG processes and methods have been updated as part of NICE’s transformation, the key message that payers require evidence linked to claimed benefits remains valid.

### Implications for manufacturers

Our findings confirm the importance of clearly articulated claims supported by specific evidence, emphasizing the need for careful planning for companies using HTA as part of their market access strategy. Further research, particularly comparative or randomized studies, was encouraged for the majority of products that were not recommended, and this is as expected, and consistent with previous studies, because the evidence generation to prove the effectiveness of medical technologies has been described as challenging due to their short lifespan (5;7;10). The need for clinical evidence supporting claims could create uncertainty for the manufacturers when the evidence is considered sufficient. There is no clear pattern in what kind of or quantity of evidence leads to a recommendation, and the implication for manufacturers is that it is difficult to predict what evidence will be deemed acceptable to NICE. It is therefore advisable to take advantage of the opportunity for scientific advice, which NICE offers (11).

### Unanswered questions and future research

As market access for medical technologies through HTA bodies is a dynamic process, future research should encompass developments in processes and methods including the increasing adoption by HTA agencies of a life-cycle approach to technology evaluation, such as NICE’s early value assessment (EVA) initiative (12). Further analyses using methods like ours of recommendation outcomes from other HTA bodies would also be helpful for technology developers. It would also be valuable to examine claims and evidence by medical technology category because, due to the time frame of our sample, digital health technologies were under-represented in this study sample. Finally, it would be informative to study the influence on recommendation outcome of other factors including company size, product type, economic evidence, and disease area (2;5;7).

## Conclusion

Between 2010 and 2023, the technology was recommended in forty-six of sixty-eight MTGs. A significant correlation was found between receiving a recommendation in the MTG program and having clinical evidence, of any quantity or quality, supporting claims. With a clear vision of which claims are made about the medical technology, the supporting evidence can be planned to support these early in the development. These considerations are important for the success of market access and the future market share of medical technology. However, the development and assessment of medical technologies is a dynamic process; therefore, future research about which features influence the decision is important.
